# Multisite electrophysiological recordings by self-assembled loose-patch-like junctions between cultured hippocampal neurons and mushroom-shaped microelectrodes

**DOI:** 10.1038/srep27110

**Published:** 2016-06-03

**Authors:** Nava Shmoel, Noha Rabieh, Silviya M. Ojovan, Hadas Erez, Eilon Maydan, Micha E. Spira

**Affiliations:** 1 The Alexander Silberman Institute of Life Science, Department of Neurobiology, The Hebrew University of Jerusalem, Edmond J. Safra Campus, Jerusalem, 91904, Israel; 2 The Harvey M. Kruger Family Center for Nanoscience, The Hebrew University of Jerusalem, Edmond J. Safra Campus, Jerusalem, 91904, Israel; 3 The C. Smith Family and Prof. J. Elkes Laboratory for Collaborative Research in Psychobiology, Department of Neurobiology, The Hebrew University of Jerusalem, Edmond J. Safra Campus, Jerusalem, 91904, Israel

## Abstract

Substrate integrated planar microelectrode arrays is the “gold standard” method for millisecond-resolution, long-term, large-scale, cell-noninvasive electrophysiological recordings from mammalian neuronal networks. Nevertheless, these devices suffer from drawbacks that are solved by spike-detecting, spike-sorting and signal-averaging techniques which rely on estimated parameters that require user supervision to correct errors, merge clusters and remove outliers. Here we show that primary rat hippocampal neurons grown on micrometer sized gold mushroom-shaped microelectrodes (gMμE) functionalized simply by poly-ethylene-imine/laminin undergo self-assembly processes to form loose patch-like hybrid structures. More than 90% of the hybrids formed in this way record monophasic positive action potentials (APs). Of these, 34.5% record APs with amplitudes above 300 μV and up to 5,085 μV. This self-assembled neuron-gMμE configuration improves the recording quality as compared to planar MEA. This study characterizes and analyzes the electrophysiological signaling repertoire generated by the neurons-gMμE configuration, and discusses prospects to further improve the technology.

Extracellular recordings by substrate integrated planar microelectrode arrays (MEAs) are considered the “gold standard” for millisecond-resolution, long-term, large-scale, cell- noninvasive electrophysiological recordings and stimulation of *in vitro* and *in vivo* neuronal networks^1^,^2^,^3^. Although planar MEA platforms are used extensively, they suffer from a low signal to noise ratio and low source resolution. These drawbacks are solved by tedious spike-detecting, spike-sorting and signal averaging techniques which rely on estimated parameters and require user supervision to correct errors, merge clusters and remove outliers^1^,^4^,^5^. Averaging of recorded field potentials (FPs) leads to loss of essential information as to the relevance of changes in FPs shapes, amplitudes and patterns over short or long experimental sessions as well as in relation to behavioral tasks. Planar electrode based MEA are “blind” to sub-threshold synaptic potentials generated by individual neurons. Neurons that do not fire action potentials are thus not “visible” to the experimenter. Since in some brain areas a large fraction of the neurons fire at very low rates or do not fire at all, their subthreshold contributions to neuronal circuit activities are undetected or ignored^6^,^7^,^8^,^9^. The neglect of silent neurons due to the technical limitations of current MEA continues despite the clear documentation that meaningful subthreshold signaling between neighboring neurons plays a critical role in neuronal network computations^10^.

In recent years a number of laboratories have started to develop a new family of MEA technologies to overcome these shortcomings^11^. These include MEA constructed by vertical micro^12^ or nano-electrodes (nanowires) that either penetrate the plasma membrane of excitable cells like sharp electrodes^7^,^13^,^14^,^15^,^16^,^17^,^18^, or nano-transistor based MEAs that are mechanically manipulated into the cells^19^,^20^,^21^,^22^. Iintracellular recordings from cultured cells by MEA technologies of vertical nanowires (nanopillars) and nanotransistors have been conducted on cultured primary cardiomyocytes^12^,^17^,^20^,^21^,^22^ cultured LH-1 cell lines^12^,^17^,^18^, the CHO cell line^7^ and HEK-293 cells^14^,^15^. Nevertheless, the dimensions, adhesion and growth patterns of primary cultured mammalian neurons and the above-mentioned cell types differ substantially. These differences may be the underlying reason why so far only one study has been published using vertical nanowire-based MEA for intracellular recordings from cultured mammalian neurons^15^. Although the technology used in this study is scalable, so far it has been used to document recordings of spontaneous action potentials from a single neuron rather than from neuronal networks. All in all, this family of nanotechnologies which generated promising results at the proof-of-concept level did not mature to provide simultaneous multisite recordings from cultured neuronal networks.

In recent years our laboratory has developed a new approach in which micrometer-sized, extracellular gold mushroom-shaped microelectrodes (gMμEs) record attenuated synaptic and action potentials exhibiting the characteristic features of intracellular recordings (the IN-CELL recording method). In these studies^23^,^24^,^25^,^26^,^27^,^28^,^29^,^30^ we demonstrated that cultured *Aplysia* neurons tightly engulf the gMμEs to form a high seal resistance. This, together with the increased conductance of the membrane patch that faces the gMμE, makes it possible to record action potentials and subthreshold synaptic potentials with qualities and biophysics similar to those obtained by perforated patch recording electrodes^31^,^32^.

Earlier attempts by our laboratory to apply the IN-CELL recording method to cultured mammalian neurons by gMμEs-MEAs were only partially successful because they were conducted using an 8 × 8 gMμE array with an electrode pitch of 20 μm. This high density electrode design was implemented with a correspondingly small 146 × 146 μm culturing surface area. As a result, the cultured rat hippocampal neurons did not develop typical network behavior and only sparse neuronal activity was monitored from one or two cells at a time^28^.

In the present study, for the first time we report on the significant progress in this gMμEs-MEA technology for multisite *in vitro* recordings from a mammalian neuronal network. This manuscript characterizes and analyzes the mechanisms that underlie the generation of the observed electrophysiological signaling repertoire and defines approaches to further improve the neurons-gMμE junction.

## Results

### Overall characterization of spontaneous electrical activity recorded by gMμE-MEA

The gMμE based MEAs used in the present study were composed of 8 × 8 gMμEs with a “mushroom-cap” diameter of 1–2 μm, a stalk diameter of 0.75–1 μm and an electrode pitch of 100 μm. Altogether the array covered a recording surface of 0.9 × 0.9 mm. The flat surface in-between the gMμEs was made of SiO_2_. The gMμE-MEAs were functionalized by poly-ethylene-imine (PEI) and laminin^33^. To increase the probability that the neuron’s cell bodies would be in close physical contact with the gMμE-caps we prepared dissociated hippocampal cells from 17 day old rat embryos^33^ at a high density of approximately 500,000 cells/ml seeding medium. 200 μl of the cells in the seeding medium were then pipetted to the center of the gMμEs-MEAs for 6 h. Thereafter 800 μl of feeding medium was added to the devices. To prevent glial cell proliferation, 2.5 μM cytosine β-D-arabinofuranoside hydrochloride (ara-c) was added to the culture medium on the 3^rd^ DIV^33^. In all experiments voltage calibration square pulses were delivered to the bathing solution through an Ag/AgCl electrode by an isolated pulse generator^26^,^27^,^28^,^29^. Recording of the activity was made with a wide band filter of 1 Hz to 10 kHz from 7–25 day old cultures.

It is important to recall that the gMμE-MEA and culturing procedures used in the present study differed from earlier studies conducted by our laboratory: (a) the spacing between the gMμE and the effective recording area was five times larger in the present study. This enabled cultured rat hippocampal neurons to form functional networks. (b) The use of embryonic hippocampal neurons rather than hippocampal cells derived from new-born rats helped to lower the density of the glial cells (approximately 3%^34^) and thereby increase the probability that the neurons would be in physical contact with the gMμE. (c) The high density seeding of dissociated cells also increased the probability that individual neurons would be in direct contact with the electrodes.

The hippocampal culture grown on gMμE-MEA revealed spontaneous activity from day 7 DIV. Bursting activity was detected from day 10 onward as described in earlier studies using substrate integrated planar MEA.

**Figure 1:**
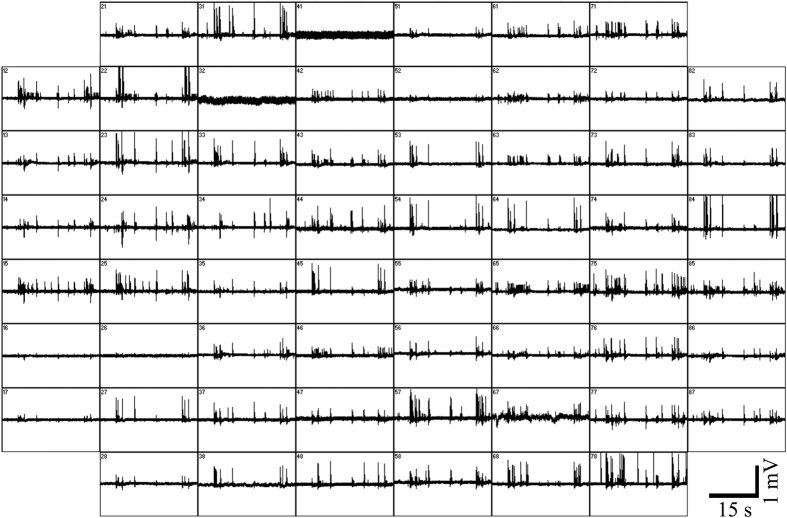
Spontaneous activity recorded by gMμE-MEA from cultured hippocampal neurons 17 DIV. Each box represents 30 s of recording from a single gMμE. Note that the majority of the gMμEs recorded monophasic positive action potentials.

**Figure 2:**
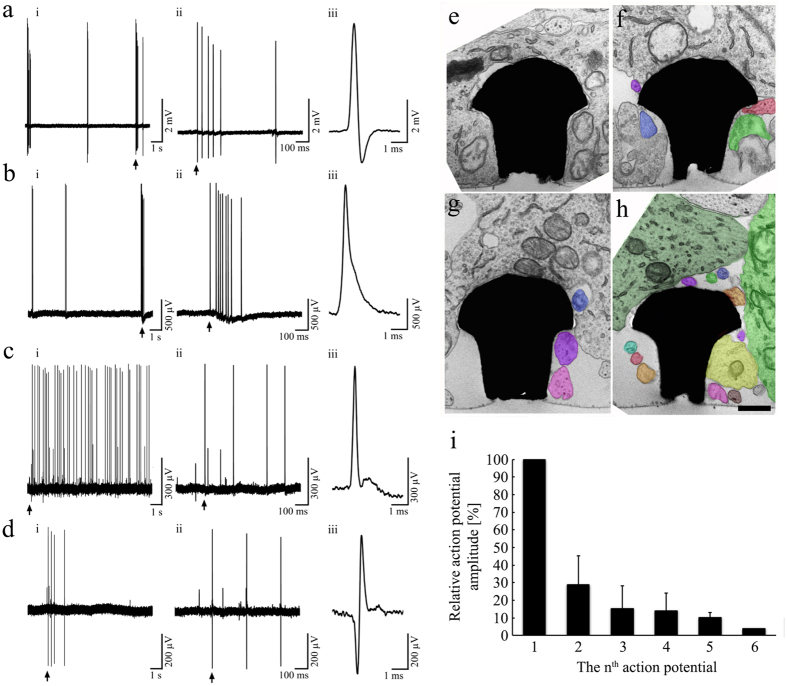
Recordings of action potentials by gMμEs. As assessed by the amplitude and shape, a gMμE can record action potentials generated by a single neuron (**a,b**) or a number of neurons or neurites (**c,d**). i, ii and iii depict the same recordings at different time scales. Multiple neurons recordings by a single gMμE may be the outcome of the structural configurations depicted in the TEM images of (**e**–**h**). Cell body profiles are shown in gray, different neurites are labeled in different colors. The cell bodies and neurites were identified using low magnification images (not shown). Because of the limited surface area of a gMμE the relative amplitude of the 2^nd^, 3^rd^, 4^th^, 5^th^ and 6^th^ neuron or neurites recorded by a single gMμE are relatively smaller with respect to the largest amplitude (**i**).

The most striking difference between the recordings of action potentials by flat MEA and gMμE-MEA is that whereas substrate integrated planar MEA record FPs dominated by negative-peak or biphasic-signals^35^ with amplitudes typically ranging from 40 to100 μV and have a signal to noise ratio (SNR) of ≤5^1^,^35^, the gMμE-MEA recordings were dominated by positive monophasic action potentials (Figs 1 and 2). It is important to note that monophasic high peak amplitudes ≥100 μV are rarely obtained using planar electrodes arrays, whereas when using the gMμE-MEA, 34.48% of the gMμEs recorded potentials ≥200 μV and 10.64% recorded potentials in the range of 500–5,085 μV (Figs 1 and 2, Supplementary Fig. 1).

Based on the spike amplitudes and their shapes, it was established that out of the total population of gMμEs that recorded signals ≥300 μV, 36.1% (n = 56) recorded APs from a single neuron, 32.3% (n = 50) recorded firing from two neurons, 20.6% (n = 32) from three neurons and the remainder 11% (n = 17) from 4–6 neurons (Fig. 2). The fact that a fraction of the gMμEs recorded from a number of neurons can be attributed to the structural relationships formed between the neurons and the gMμE. Transmission electron microscope images of the neuron-gMμE interfaces revealed that a single gMμE with a cap diameter of approximately 2 μm can be totally or partially engulfed by a single neuron cell body or be in contact with a cell body and a number of neurites (Fig. 2e–h). Among other parameters, the relative contact surface area formed between a neuron and a gMμE and the cleft width determine the electrical coupling coefficient levels between the neurons and gMμEs^26^,^27^,^30^,^34^,^36^,^37^.

Consistent with the above, for gMμEs that recorded from two neurons (evaluated by the spike shapes and amplitudes) the relative average amplitude of the smaller spike amplitude was 28.9 ± 16.4% of the largest spike. In gMμE that recorded from three neurons (or three compartments of the same neuron) the average amplitude of the third spike was found to be 15.5 ± 12.8% of the largest one and the fourth to be 14.2 ± 9.8% of the largest signal (Fig. 2i). Since the large and small action potentials recorded by a single gMμE are not always time-locked and bursts of small and large action potentials may appear independently of each other (Supplementary Fig. 2), it is reasonable to assume that in these cases the activity is generated by different neurons that make different levels of physical contact with a single gMμE rather than by different compartments of the same neuron (Fig. 2e–h). Computer simulations of the neuron-gMμE junction by Ojovan *et al*.^34^ revealed that it is possible to record APs from neurons that only partially engulf a single gMμE.

**Figure 3:**
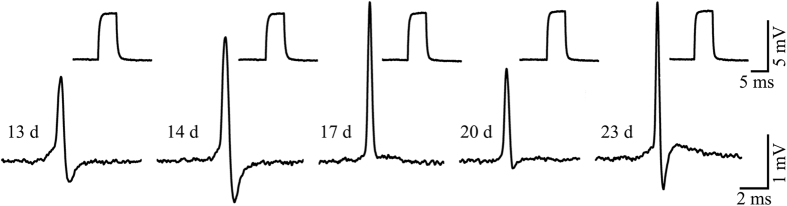
Changes in the shape and amplitudes of spontaneous action potentials recorded by one gMμE over a period of 10 days. Note that the amplitude and shape of the calibration pulses did not change, indicating that the electrode properties remained unaltered during the experiment.

Examination of the changes in the shapes and amplitudes of spikes recorded by individual gMμE over time in culture revealed that as in planar sensor MEAs, the FP shape and amplitude change over days in culture^35^. Whereas the amplitude of a spike recorded by a gMμE may gradually increase, decrease or abruptly disappear over consecutive days, the overall positive monophasic shape is maintained (Fig. 3). The changes in AP shapes and amplitudes may reflect changes in the engulfment level and/or the cleft width formed between the neuron and the gMμE due to changes in the neuron’s shape, the macro or micro-neuron’s movement with respect to the electrode, and neuronal death. It is also conceivable that alterations in ionic channel expression, distribution and density within the junctional membrane may underlie some of these changes (see below for further discussion).

### The repertoire of electrophysiological signals recorded by individual gMμEs

At first glance, the monophasic, positive peak that dominated the spontaneous action potential recordings by the gMμE-MEA resemble the IN-CELL recordings of attenuated action potentials obtained from cultured *Aplysia* neurons^26^,^27^. Nevertheless, the recorded APs differed from IN-CELL recordings in their time course. At 50% AP peak height the average duration of APs with amplitudes ≥300 μV was 0.47 ± 0.28 ms (n = 154). Sample recordings of intracellular action potentials by patch electrodes from a 14 day old culture revealed that at 50% peak height the spike duration was longer in the range of 1–4 ms^38^. The amplitudes and characteristic short duration of the recorded potentials by the gMμE (Figs 2 and 3) led us to surmise that under the culture condition used in the present study the engulfment of the gMμE formed a loose seal-like (juxtacellular) configuration^39^,^40^ rather than an IN-CELL recording configuration (for a biophysical explanation of the differences between juxtacellular and IN-CELL recording see next paragraph). A loose seal/juxtacellular recording configuration is often used under *in vivo* conditions to improve the recorded spike amplitudes and source separation by single patch electrodes. In these conditions the juxtacellular recording is generated by micromanipulation of a patch electrode against the neuron’s plasma membrane and/or by suctioning the membrane into it.

To examine whether the above hypothesis could account for the shape, amplitudes and duration of the recorded APs, we next simulated the hippocampal neuron-gMμE configuration.

### Simulations of single action potentials

**Figure 4:**
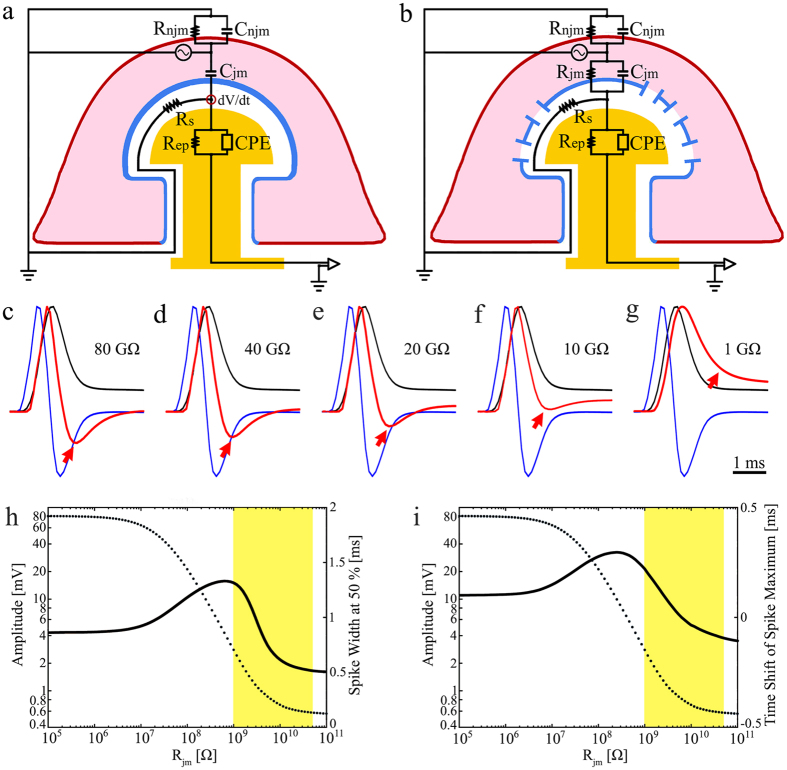
The analog electrical circuit of a neuron-gMμE junction, and an estimate of the impact of the junctional membrane properties on the input/output relationship of the junction. The neuron’s (pink) plasma membrane is subdivided into a non-junctional membrane (*njm*, red) that faces the culture medium, and a junctional membrane (*jm*, blue) that faces the electrode. Both the *njm* and the *jm* are represented by a resistor and capacitor in parallel *R*_*njm*_, *C*_*njm*_, *R*_*jm*_ and *C*_*jm*_ respectively. The cleft formed between the plasma membrane and the gMμE (white) is represented by a resistor (*R*_*s*_). The gMμE is represented by a constant phase element (CPE) and a resistor in parallel (*R*_*ep*_)^47^. (**c–g**) Simulation of the shape, amplitude, AP width, and AP peak time as a function of the junctional membrane resistance. The normalized input AP (black), its calculated time derivative (blue) and the simulated output (red) for the indicated *R*_*jm*_ values 80–1 GΩ. The shape of the output APs (red) changes (red arrow) from being similar to the time derivative of the input AP (**c**), to an intracellular recording (**f,g**). Aside from the dependence of the simulated output shape, the increase in *R*_*jm*_ value is associated with a decrease in the amplitude of the simulated output AP (h and i-dashed line), a change in the simulated AP duration (**h**, -black line), and a shift in the AP peak time with respect to the input AP (**i**, black line). The expected amplitude, duration and peak time of the simulated APs in the range of *R*_*jm*_ 1–40 GΩ are shaded yellow.

The simulation was conducted using the SPICE simulation system (Tanner EDA v.15) of passive analog electrical circuits of the junction formed between a neuron and a gMμE (Fig. 4a,b)^26^,^27^,^28^,^29^,^34^,^41^. The circuits illustrate two operational modes of the membrane facing the gMμE (the junctional membranes). In Fig. 4a, we assumed that the junctional membrane resistance (R_jm)_ was very large (>100 GΩ)^26^,^27^,^34^; thus the resistive component of the junctional membrane could be neglected and the membrane represented by a capacitor (C_jm_) with a value that corresponded to its surface area times 1 μF/cm^2^. This together with the seal resistance formed by the cleft between the plasma membrane and the gMμE (R_s_) configured a passive electrical differentiator that generated an output potential proportional to the time derivative of the input^42^. By contrast, the circuit shown in Fig. 4b assumed that R_jm_ was low (~1 GΩ)^26^,^27^. As a result, the circuit properties were transformed from a differentiator (Fig. 4a) to an element that did not distort the shape of the wave form (Fig. 4b). The change in the electrical properties of the analog electrical circuit (Fig. 4a,b) corresponded to the transition between a loose seal/juxtacellular recording configuration and an IN-CELL recording. In fact, a gradual change in the relationships between the junctional membrane resistance (R_jm_) and capacitance (C_jm_) is expected to generate a spectrum of outputs ranging from the juxtacellular to the IN-CELL recording modes. This spectrum of outputs is illustrated in the simulations shown in Fig. 4c–i.

The simulations in Fig. 4 were conducted using the following parameters: (i) The neuron’s input resistance from which the junctional membrane resistance was derived was set at 100 MΩ^43^. (ii) a membrane capacitance of 1 μF/cm^2^, (iii) a seal resistance (R_s_) 50 MΩ^27^,^33^, (iv) an electrode CPE at 1 KHz, an electrode parallel resistance (25 MΩ and 10 MΩ respectively for details see Supplemental Material) and (v) an amplifier impedance of 20 MΩ at 1 kHz. Based on the above parameters, Fig. 4c–g illustrates the effect of changing the relationships between *R*_*jm*_ *and C*_*jm*_, in terms of the shape, amplitude and duration of the simulated APs. In the figure we superimposed the normalized peak amplitudes of the input potential (black traces), the calculated time derivative of the input AP (blue traces) and the simulated output AP (red traces). Clearly, as the *R*_*jm*_ values decreased, the shape of the output AP transforms from a potential that resembles the time derivative of the input AP (Fig. 4c compare the red and blue traces) to a monophasic shaped output AP that more closely resembles the input AP (Fig. 4g, compare the red and black traces). The transformation of the shape of the AP is associated with an increase in the AP duration (Fig. 4c–h, black line) and the shift in the AP peak time with respect to the peak of the input AP (Fig. 4c–g,i, black line).

Comparing the actual AP recorded in the experiments to the simulations suggests that the experimentally recorded APs with amplitudes in the range of 300–1000 μV and a duration of 0.44 ± 0.1 ms (at 50% height) correspond to the simulated action potentials generated by neurons with an *R*_*jm*_ value in the range of 20 GΩ when *R*_*s*_ is set to be 50 MΩ.

It should be noted that the amplitude of the output AP can be improved by increasing the seal resistance (not shown). Theoretically, an increase in the gMμE surface area is also expected to increase the AP amplitude. Nevertheless, as shown by Ojovan *et al*.^34^, the innate cell biology of rat hippocampal neurons limit the engulfment of gMμE to a cap diameter of 2–2.5 μm.

### Simulation of the characteristic decline of action potential amplitudes within bursts and the potential to record synaptic activity

**Figure 5:**
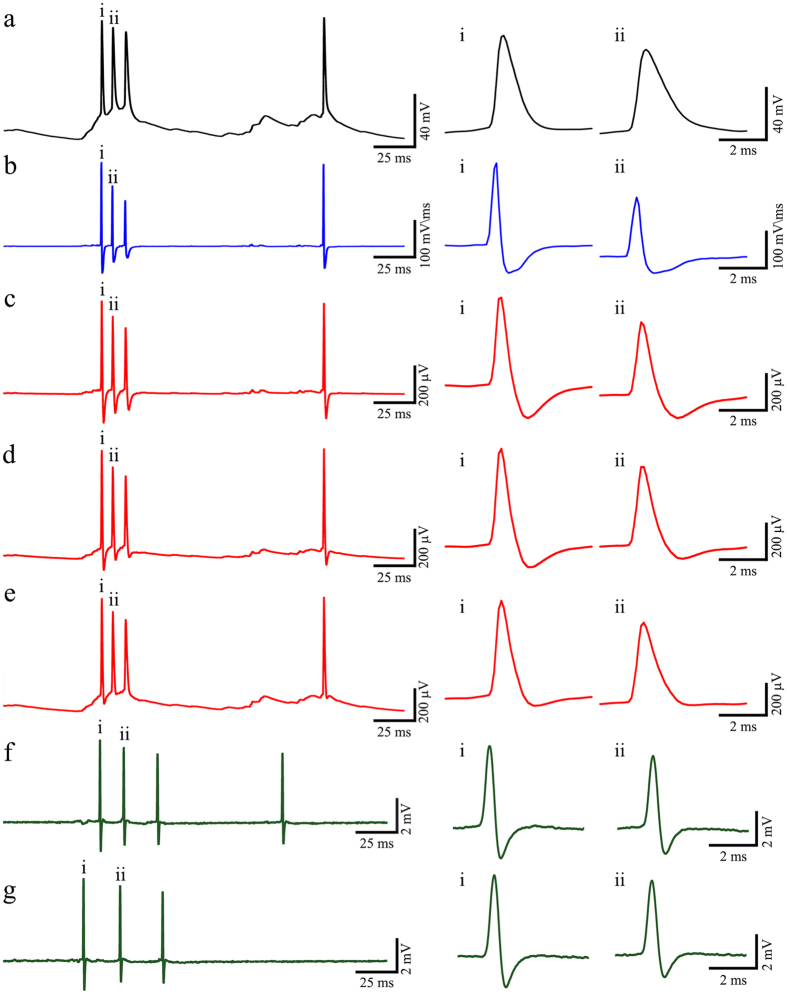
Simulation of the characteristic decline in AP amplitude within a burst and the potential to record a barrage of post synaptic potentials by the gMμE-MEA. For the simulation a trace of patch electrode recording of APs and a barrage of synaptic potential (**a**) were fed into a simulation circuit. The circuit parameters were set to be: *R*_*s*_ - 50 MΩ, *R*_*ep*_- 10 MΩ, CPE 25 MΩ at 1 KHz and the amplifiers’ impedance −20 MΩ at 1 KHz. (**b**) The calculated time derivative of the bursts (blue). Simulations of the output at *R*_*jm*_ values of (**c**) 80 GΩ, (**d**) 20 GΩ, (**e**) 10 GΩ (red). (**f–g**) Two examples of actual AP bursts recorded by a gMμE-MEA (green). The time scale of the APs labeled by i and ii within the trains is enlarged on the right hand side showing the corresponding i and ii APs. Note that the accentuated declines in AP amplitude in the calculated time derivative (**b**) and in the simulation model (**c–e**) are related to the slowdown of the AP rise time recorded by the patch electrode in (**a**). The shape (duration and amplitude) of the recorded action potentials in (**f,g**) are similar to the recordings in (**b**,**c**). It is worth noting that in the simulations synaptic potentials are detected when *R*_*jm*_ is set at 20 and 10 GΩ. The very small ripples in the actual recordings (**f,g**) might also be synaptic potentials.

Recordings of trains of APs by gMμE are characterized by consecutive or abrupt drops in the AP amplitudes by up to ~35% (for example, Fig. 2a,b). Moderate decreases in AP amplitudes within a burst were also observed by intracellular recordings. The differences in the rate and extent of the AP amplitude decline when recorded by a patch electrode and a gMμE (compare Fig. 5a,f,g) can be attributed to the fact that under the current experimental conditions, the shapes of the APs are altered by the passive electrical differentiator properties of the neuron-gMμE junction (as discussed above, Fig. 4). It should be noted that the rise and decay times of the APs within bursts are slowed down (see AP i and ii in Fig. 5a). As a result , the time derivative of this burst yields a pronounced diminution in the AP amplitude (Fig. 5b). The effect of *R*_*jm*_ on the shape and amplitude of the output APs are illustrated in Fig. 5 for *R*_*jm*_ of 80, 20 and 10 GΩ (Fig. 5c–e, respectively). For comparison purposes, Fig. 5f,g depicts actual recordings of short AP bursts from gMμE. Comparison of these APs to the calculated time derivative of the input AP (Fig. 5b, blue trace) and the simulations (Fig. 5c–e, red traces) suggests that these APs were experimentally recorded through 80–100 GΩ high *R*_*jm*_.

Note that in the simulation in Fig. 5 the integrated envelope of postsynaptic potentials that are fed into the analog electrical circuit (Fig. 5a, black trace) are hardly detected in the time derivative traces of the input (Fig. 5b, blue). This PSP activity is nevertheless detected in the simulations when the value of *R*_*jm*_ is in the range of 10–20 GΩ as shown in Fig. 5d,e, respectively. Although we noted a “rippling in the traces” in a number of recordings prior or between bursts of APs we cannot unequivocally ascribe these to the recordings of synaptic activity, as discussed below.

### Recordings of synaptic potentials?

**Figure 6:**
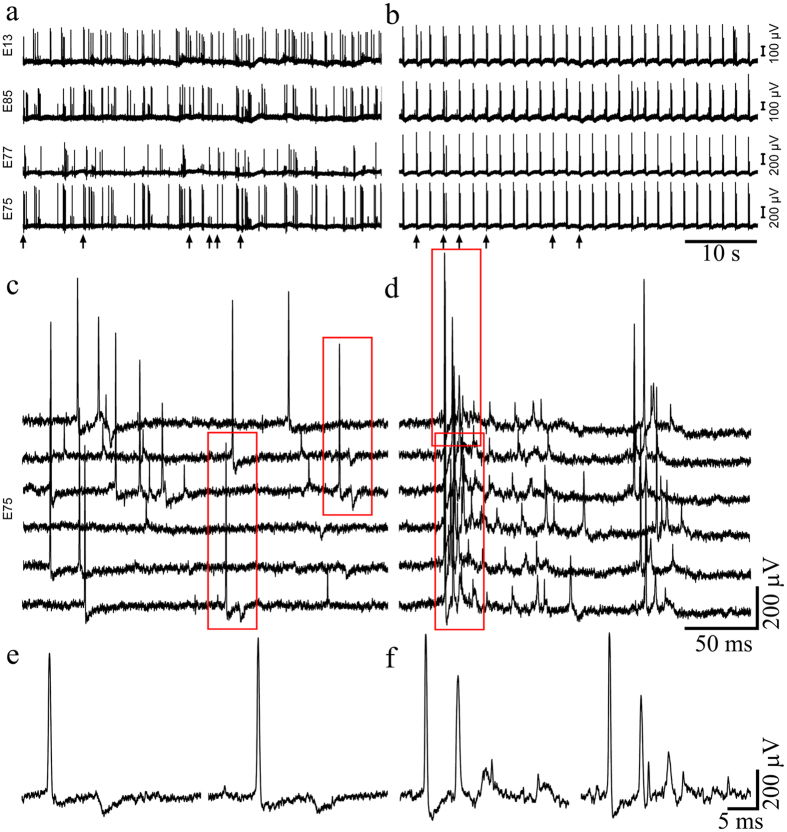
The effect of GABAzine on spontaneous spike patterns and the recorded electrophysiological signaling repertoire. (**a**) spontaneous firing as recorded by 4 gMμE from neurons at 19 DIV. (**b**) Recording from the same electrodes 10 min after the application of 10 μM GABAzine to the culture medium. (**c,d**) Enlargements of the bursts indicated by arrows from electrode E75 in (**a**,**b**) respectively. Note that electrode E75 recorded large and small spikes (**a**). (**e,f**) Enlargements of the potentials enclosed by red boxes in (**c**,**d**) respectively. Note that GABAzine application changed the firing pattern to bursts in which the large and small spikes are more synchronized. (**e**) Low amplitude, long duration negative potentials recorded before GABAzine application. These potentials disappear after GABAzine application, and positive, low amplitude, long duration potentials appear (**f**).

In a small number of experiments we observed the presence of low amplitude (~100 μV) negative or positive potentials with slower rise and decay times (ripples). Given the recent finding that field potentials generated by single neurons decay to a third of their amplitude within a distance of approximately 100 μm^38^, it is possible that these potentials could reflect the pickup of FPs generated by neuronal clusters within ~300 μm from the recording gMμE (Fig. 6 and Supplementary Fig. 2). Alternatively, these potentials could reflect a barrage of high amplitude postsynaptic potentials (Fig. 5). Currently we cannot differentiate between these possibilities by rigid biophysical criteria such as by shifting the membrane potentials of the cells that generate these signals in an attempt to reverse the potential. In this respect, it is interesting to note that bath application of the GABAergic postsynaptic blocking reagent GABAzine (1–10 μM, Sigma Aldrich) to cultures resulted in (a) the transformation of the firing pattern into discrete regular bursts (Fig. 6a,b, respectively ) and (b), the disappearance of the slow negative potentials. It is conceivable that if the slow low amplitude potentials had been generated by synchronized bursts of APs generated by remote neuronal clusters, the frequency and amplitude of the negative potentials could have increased rather than disappeared.

The simulations in Fig. 5 suggested that it is physically possible to record postsynaptic potentials given that the junctional membrane resistance is in the range of 10–20 GΩ and R_s_  = 50 MΩ. Because of the biological nature of the seal and junctional membrane resistances, both parameters can vary and are not precisely known^28^,^34^. Therefore, to better assess the physical limits of the neuron-gMμE junction to transfer detectable synaptic potentials we extended (supplemental Fig. 3) the range of the simulated R_s_ and R_jm_. These simulations suggest that R_jm_ values in the range of ≤10 GΩ, and seal resistances ≥50 MΩ suffice to permit recordings of synaptic potentials with a source amplitude of 10 mV. R_jm_ ≥ 10 GΩ, and R_s_< 50 MΩ, would not permit recording of a 10 mV PSPs. For a R_jm_ of 100 GΩ even Rs of >100 MΩ would be insufficient to permit recordings of a 10 mV PSP.

In summary, although far from being conclusive, taken together the results (electrophysiological, pharmacological and simulations) are consistent with the possibility that the relatively slow low amplitude potentials recorded by the gMμE could represent large (10 mV) synaptic potentials.

## Discussion

gMμE-MEA functionalized by a conventional PEI and laminin is sufficient to promote a “self-assembly” process that leads to the formation of a neuron/gMμE loose patch-like configuration. The engulfment of the gMμE by the neurons enables multisite, high quality recordings of monophasic positive APs from many cultured mammalian neurons comprising a functional network. Simulations of neuron-gMμE models (Fig. 4) suggest that the loose patch recording configuration can be transformed into an IN-CELL recording by decreasing *R*_*jm*_. This could theoretically be done by: (a) using gMμE with a larger cap-diameter. In these conditions the engulfment of gMμE with a larger surface area would be associated with a larger junctional membrane surface and decreased *R*_*jm*_. Unfortunately, the innate cell biological properties of cultured rat hippocampal neurons limit the ability of the neurons to engulf gMμE-cap with a diameter of 2–2.5 μm^34^. Therefore, this solution cannot be applied for practical purposes. Increasing the roughness (surface area) of the gMμE surface without increasing the size of the electrode could also lead to a small increase in the effective surface area of the junctional membrane and improve the recording quality. (b) An alternative mechanism would be to directly modulate the junctional membrane properties. It is generally believed that the specific capacitance of a biological membrane (1 μF/cm^2^) cannot be practically modulated. However, the junctional membrane resistance can be reduced by localizing voltage independent ion channels into it^26^,^27^,^30^ by electroporation^15^,^16^,^17^,^18^ or by insertion of exogenous nanopores^31^,^32^,^44^. For example, we estimated that the insertion of 10 voltage independent potassium channels with a conductance of 10–100 pS would be sufficient to reduce the resistance of a 10 μm^2^ junctional membrane from 100 GΩ to 10–1 GΩ. This modification could be sufficient to enable intracellular recordings of minimally filtered APs and synaptic potentials (Fig. 5f,g). We believe that the results presented in this study already demonstrate the advantages of gMμE-MEA over planar-MEA and provide the foundation to further develop the gMμE methodology for IN-CELL recordings from mammalian neurons.

## Materials and Methods

### Fabrication of the gold mushroom shaped microelectrode array

gMμE were prepared on 300 μm thick glass wafers (AF45 Schott Glass) by means of photolithography and electroplating techniques. Briefly, the wafers were coated with a Ti (10 nm)/Au layer (100 nm) by way of thermal evaporation, spin-coated with photoresist AZ-1505 (4,000 RPM) and hard baked for 2 min (120 °C). Thereafter a first photolithographic process to define the conducting lines was performed by wet etching of the Ti/Au in between the conducting lines. Next, a second lithographic step using Shipley S-1813G2 photoresist (4,000 RPM) hard baked for 10 min (120 °C) was performed to open up 0.9 μm holes for the electro deposition of the gMμE-stalks. A similar procedure was used to open up the contact pads. Then, the gMμEs were formed by gold electroplating at a current density of 0.2 mA/cm^2^ for 3 h. The photoresist layer was stripped off and a layer of silicon nitride (150 Å)/silicon oxide (3,000 Å) was deposited by chemical vapor deposition. This layer serves as an encapsulation layer for the conducting electrode lines. A third layer of photoresist was then photolithgraphically patterned, followed by wet silicon nitride and silicon oxide etching to selectively remove the silicon nitride and silicone oxide from the contact pads and the mushroom caps. The photoresist layer was then stripped using acetone and isopropanol. The wafers were then diced and underwent manual bonding to 60 pad printed circuit boards to which a glass ring with a diameter of 20 mm was attached to create a cell culture chamber.

### Surface functionalization

Fabricated gMμE-MEAs were washed and sterilized by incubation in 70% ethanol for 2 h. Then, the ethanol was rinsed with double distilled water and functionalized by 0.1 mg/ml PEI (Sigma–Aldrich) and 25 μg/ml laminin (Sigma–Aldrich) in 0.1 M sodium borate, 10 mM HEPES solution (pH = 8.2) for 12 h prior to cell seeding.

### Cell culture

Primary rat hippocampal neurons were obtained from 17 d old embryos, as described by Kaech and Banker^32^. Briefly, a pregnant WT (Sprague Dawley) female rat was deeply anesthetized with isoflurane, the embryos removed and decapitated. The embryonic hippocampi were dissected out and treated with papain solution (1.5 mM CaCl_2_, 0.5 mM EDTA and 18 μl/ml papain (16–40 units/mg protein), 20 mM HEPES (Sigma–Aldrich) in Hanks Balanced Salt Solution (HBSS- Biological industries) at 7.4 pH) for 45 min., and were serially and gently triturated once every 10 min. The papain solution was washed away by a seeding medium (Neurobasal with 5% FBS, 2% B27, 1% GlutaMAX (Life technologies), 1% Penicillin-Streptomycin Amphotericin B Solution (Biological Industries)). The neurons were then triturated again in the seeding medium and concentrated to approximately 500,000 cells/ml. 200 μl of the cell-seeding medium containing the neurons was then placed in the center of PEI-laminin functionalized gMμEs-MEAs for 12 h. Thereafter, 800 μl of serum-free maintenance/feeding medium (Neurobasal electro medium, 2% B27 electro, 1% GlutaMAX, 1% Penicillin-Streptomycin Amphotericin B Solution) was added. Three days after seeding 2.5 μM ara-c (Sigma–Aldrich) was added to prevent glial cell proliferation. Half of the maintenance medium was replaced every 3–5 days by new feeding medium. Hippocampal cultured cells were kept at 37 °C in a humidified atmosphere of 5% CO_2_. Cultures were kept up to 25 DIV. All procedures were approved by the Committee for Animal Experimentation at the Institute of Life Sciences of the Hebrew University of Jerusalem. All procedures (methods) were carried out in accordance with the approved guidelines.

### Electrophysiology

gMμE-MEA devices were amplified by an AC, 60-channel amplifier (MEA-1060-Inv-BC, MCS) with frequency limits of 1–10,000 Hz. and a sampling rate of 10 kHz. Recordings were carried out at 37 °C in the culturing medium. The number of spikes recorded by a single gMμE was determined manually. Measurements of spike amplitudes and durations were done using Clampfit software (version 10.4.0.36). To examine for possible crosstalk between gMμE-MEA channels we averaged the voltage amplitude of (5–10) synchronized individual channels in the vicinity of a channel from which a large spike was read (100–200 μm) (supplemental Fig. 4). A sampling of 70 clusters revealed no detectable crosstalk.

### Electron microscopy

For TEM analysis, cells cultured on matrices of gold mushroom microprotrusions were fixed, dehydrated and embedded in Agar 100 within culture dishes constructed of matrices of protruding gold mushroom shaped protrusions as previously described^34^,^45^.

### Computer simulation

Computer simulations were conducted using SPICE (Tanner EDA v.15), as well as the passive analog electrical circuit depicting a gMμE interfaced with a neuron as shown in Fig. 4 and detailed by Ojovan *et al*.^34^. Calculations and graph presentations were made using MATLAB (20014A). For the simulations the mushroom shaped protruding structure was constructed of an ellipsoid-shaped cap with a height of 0.5 μm, a diameter of 2 μm and 1 μm-high cylindrical stalks with a diameter of 1 μm. The total surface area of the electrode was calculated to be 9.8 μm^2^. For the simulations we rounded off the number to 10 μm^2^ .The detailed calculations of the gMμE surface area are given in Ojovan *et al*.^34^.

#### Seal resistance (*R*_*s*_)

The simulations conducted in the present study used seal resistances of 1–200 MΩ. In the simulation in Fig. 5 we used a seal resistance of 50 MΩ, a value that was estimated in an earlier study by Fendyur *et al*.^28^.

#### Junctional membrane resistance (*R*_*jm*_)

Using the calculated surface area of the gMμE, the corresponding surface area of the junctional membrane and the junctional membrane resistance and capacitance were estimated. A non-junctional membrane resistance of 100–250 MΩ (*R*_*njm*_) has been experimentally measured^43^,^46^. Assuming that the surface area of a cultured hippocampal neuron is approximately 6 × 10^4^ μm^2^, the resistance of a 10 μm^2^ junctional membrane patch is >100 GΩ^26^,^27^. In earlier studies it was argued that the actual resistance of the junctional membrane is significantly smaller^26^,^27^. Because of the small surface area of the junctional membrane, *R*_*jm*_ can vary substantially by the recruitment or depletion of single ion channels or by the formation of nanopores due to mechanical tension generated at the neuron-electrode interface. In the simulations in the present study we examined *R*_*jm*_ values ranging from a single MΩ to a hundred GΩ.

The junctional membrane capacitance (*C*_*jm*_) was calculated for a given contact surface area (between the simulated cells and the simulated gMμE) by multiplying the universal value of the specific membrane capacitance (1 μF/cm^2^) and the surface area.

#### gMμE resistance and capacitance

For the simulations we used gMμE depicted by two elements: a constant phase element (*CPE*) and a parallel resistor (*R*_*ep*_) (Fig. 4). The value of the *CPE* impedance was 25 MΩ at 1 KHz, and that of the *R*_*ep*_ 10 MΩ (for details see Supplemental text and Supplemental Fig. 5).

An amplifier input capacitance of 8 pF and a parallel resistance of 100 GΩ were used in all simulations.

#### Simulation of synaptic- and action-potentials

Voltage pulses were delivered to the simulated neurons between the junctional (*jm*) and non-junctional membranes (*njm*).

## Additional Information

**How to cite this article**: Shmoel, N. *et al*. Multisite electrophysiological recordings by self-assembled loose-patch-like junctions between cultured hippocampal neurons and mushroom-shaped microelectrodes. *Sci. Rep.* **6**, 27110; doi: 10.1038/srep27110 (2016).

## Supplementary Material

Supplementary Information

## References

[b1] Obien M. E., Deligkaris K., Bullmann T., Bakkum D. J. & Frey U. Revealing neuronal function through microelectrode array recordings. Front Neurosci 8, 423, doi: 10.3389/fnins.2014.00423 (2014). 25610364 PMC4285113

[b2] Massobrio P., Tessadori J., Chiappalone M. & Ghirardi M. *In vitro* studies of neuronal networks and synaptic plasticity in invertebrates and in mammals using multielectrode arrays. Neural Plast 2015, 196195, doi: 10.1155/2015/196195 (2015). 25866681 PMC4381683

[b3] Buzsaki G. . Tools for probing local circuits: high-density silicon probes combined with optogenetics. Neuron 86, 92–105, doi: 10.1016/j.neuron.2015.01.028 (2015). 25856489 PMC4392339

[b4] Quiroga R. Q., Nadasdy Z. & Ben-Shaul Y. Unsupervised spike detection and sorting with wavelets and superparamagnetic clustering. Neural Comput 16, 1661–1687, doi: 10.1162/089976604774201631 (2004). 15228749

[b5] Einevoll G. T., Franke F., Hagen E., Pouzat C. & Harris K. D. Towards reliable spike-train recordings from thousands of neurons with multielectrodes. Curr Opin Neurobiol 22, 11–17, doi: 10.1016/j.conb.2011.10.001 (2012). 22023727 PMC3314330

[b6] Epsztein J., Brecht M. & Lee A. K. Intracellular determinants of hippocampal CA1 place and silent cell activity in a novel environment. Neuron 70, 109–120, doi: 10.1016/j.neuron.2011.03.006 (2011). 21482360 PMC3221010

[b7] Aalipour A., Xu A. M., Leal-Ortiz S., Garner C. C. & Melosh N. A. Plasma membrane and actin cytoskeleton as synergistic barriers to nanowire cell penetration. Langmuir 30, 12362–12367, doi: 10.1021/la502273f (2014). 25244597

[b8] Barth A. L. & Poulet J. F. Experimental evidence for sparse firing in the neocortex. Trends Neurosci 35, 345–355, doi: 10.1016/j.tins.2012.03.008 (2012). 22579264

[b9] Shoham S., O’Connor D. H. & Segev R. How silent is the brain: is there a “dark matter” problem in neuroscience? J Comp Physiol A Neuroethol Sens Nneural Behav Physiol 192, 777–784, doi: 10.1007/s00359-006-0117-6 (2006). 16550391

[b10] Lefler Y., Yarom Y. & Uusisaari M. Y. Cerebellar inhibitory input to the inferior olive decreases electrical coupling and blocks subthreshold oscillations. Neuron 81, 1389–1400, doi: 10.1016/j.neuron.2014.02.032 (2014). 24656256

[b11] Angle M. R., Cui B. & Melosh N. A. Nanotechnology and neurophysiology. Curr Opin Neurobiol 32, 132–140, doi: 10.1016/j.conb.2015.03.014 (2015). 25889532

[b12] Braeken D. . Open-cell recording of action potentials using active electrode arrays. Lab Chip 12, 4397–4402, doi: 10.1039/c2lc40656j (2012). 22930315

[b13] Lin Z. C. & Cui B. Nanowire transistors: room for manoeuvre. Nat Nanotechnol 9, 94–96, doi: 10.1038/nnano.2014.10 (2014). 24496276

[b14] Angle M. R., Wang A., Thomas A., Schaefer A. T. & Melosh N. A. Penetration of cell membranes and synthetic lipid bilayers by nanoprobes. Biophys J 107, 2091–2100, doi: 10.1016/j.bpj.2014.09.023 (2014). 25418094 PMC4223211

[b15] Robinson J. T. . Vertical nanowire electrode arrays as a scalable platform for intracellular interfacing to neuronal circuits. Nat Nanotechnol 7, 180–184, doi: 10.1038/nnano.2011.249 (2012). 22231664 PMC4209482

[b16] Angle M. R. & Schaefer A. T. Neuronal recordings with solid-conductor intracellular nanoelectrodes (SCINEs). PLoS One 7, e43194, doi: 10.1371/journal.pone.0043194 (2012). 22905231 PMC3419643

[b17] Lin Z. C., Xie C., Osakada Y., Cui Y. & Cui B. Iridium oxide nanotube electrodes for sensitive and prolonged intracellular measurement of action potentials. Nat Commun 5, 3206, doi: 10.1038/ncomms4206 (2014). 24487777 PMC4180680

[b18] Xie C., Lin Z., Hanson L., Cui Y. & Cui B. Intracellular recording of action potentials by nanopillar electroporation. Nat Nanotechnol 7, 185–190, doi: 10.1038/nnano.2012.8 (2012). 22327876 PMC3356686

[b19] Qing Q. . Free-standing kinked nanowire transistor probes for targeted intracellular recording in three dimensions. Nat Nanotechnol 9, 142–147, doi: 10.1038/Nnano.2013.273 (2014). 24336402 PMC3946362

[b20] Gao R. . Outside looking in: nanotube transistor intracellular sensors. Nano Letters 12, 3329–3333, doi: 10.1021/nl301623p (2012). 22583370 PMC3374901

[b21] Tian B. . Three-dimensional, flexible nanoscale field-effect transistors as localized bioprobes. Science (New York, N.Y.) 329, 830–834, doi: 10.1126/science.1192033 (2010). PMC3149824 20705858

[b22] Duan X. . Intracellular recordings of action potentials by an extracellular nanoscale field-effect transistor. Nat Nanotechnol 7, 174–179, doi: 10.1038/nnano.2011.223 (2012). 22179566 PMC3293943

[b23] Spira M. E. . Improved neuronal adhesion to the surface of electronic device by engulfment of protruding micro-nails fabricated on the chip surface. Transducers ′07 & Eurosensors Xxi, Digest of Technical Papers, Vols 1 and 2, U628–U6292616 (2007).

[b24] Hai A. . Spine-shaped gold protrusions improve the adherence and electrical coupling of neurons with the surface of micro-electronic devices. J R Soc Interface 6, 1153–1165, doi: 10.1098/rsif.2009.0087 (2009). 19474080 PMC2817159

[b25] Hai A. . Changing gears from chemical adhesion of cells to flat substrata toward engulfment of micro-protrusions by active mechanisms. J Neural Eng 6, 066009, doi: 10.1088/1741-2560/6/6/066009 (2009). 19918108

[b26] Hai A., Shappir J. & Spira M. E. Long-term, multisite, parallel, in-cell recording and stimulation by an array of extracellular microelectrodes. J Neurophysiol 104, 559–568 (2010). 20427620 10.1152/jn.00265.2010

[b27] Hai A., Shappir J. & Spira M. E. In-cell recordings by extracellular microelectrodes. Nat Methods 7, 200–202 (2010). 20118930 10.1038/nmeth.1420

[b28] Fendyur A., Mazurski N., Shappir J. & Spira M. E. Formation of Essential Ultrastructural Interface between Cultured Hippocampal Cells and Gold Mushroom-Shaped MEA- Toward “IN-CELL” Recordings from Vertebrate Neurons. Front Neuroeng 4, 1–14, doi: 10.3389/fneng.2011.00014 (2011). 22163219 PMC3233721

[b29] Fendyur A. & Spira M. E. Toward on-chip, in-cell recordings from cultured cardiomyocytes by arrays of gold mushroom-shaped microelectrodes. Front Neuroeng 5, 21, doi: 10.3389/fneng.2012.00021 (2012). 22936913 PMC3426852

[b30] Spira M. E. & Hai A. Multi-electrode array technologies for neuroscience and cardiology. Nat Nanotechnol 8, 83–94, doi: 10.1038/nnano.2012.265 (2013). 23380931

[b31] Akaike N. & Harata N. Nystatin perforated patch recording and its applications to analyses of intracellular mechanisms. Jap J Physiol 44, 433–473, doi: 10.2170/jjphysiol.44.433 (1994). 7534361

[b32] Horn R. & Marty A. Muscarinic activation of ionic currents measured by a new whole-cell recording method. Journal Gen Physiol 92, 145–159 (1988). 2459299 10.1085/jgp.92.2.145 PMC2228899

[b33] Kaech S. & Banker G. Culturing hippocampal neurons. Nat Protoc 1, 2406–2415, doi: 10.1254/fpj.119.163 (2006). 17406484

[b34] Ojovan S. M. . A feasibility study of multi-site,intracellular recordings from mammalian neurons by extracellular gold mushroom-shaped microelectrodes. Sci Rep 5, 14100, doi: 10.1038/srep14100 (2015). 26365404 PMC4568476

[b35] Nam Y. & Wheeler B. C. *In vitro* microelectrode array technology and neural recordings. Crit Rev Biomed Eng 39, 45–61 (2011). 21488814 10.1615/critrevbiomedeng.v39.i1.40

[b36] Cohen A., Shappir J., Yitzchaik S. & Spira M. E. Reversible transition of extracellular field potential recordings to intracellular recordings of action potentials generated by neurons grown on transistors. Biosens Bioelectron 23, 811–819 (2008). 17959368 10.1016/j.bios.2007.08.027

[b37] Jenkner M. & Fromherz P. Bistability of membrane conductance in cell adhesion observed in a neuron transistor. Phys Rev Lett 79, 4705–4708 (1997).

[b38] Weir K., Blanquie O., Kilb W., Luhmann H. J. & Sinning A. Comparison of spike parameters from optically identified GABAergic and glutamatergic neurons in sparse cortical cultures. Front Cell Neurosci 8, 460, doi: 10.3389/fncel.2014.00460 (2014). 25642167 PMC4294161

[b39] Joshi S. & Hawken M. J. Loose-patch-juxtacellular recording *in vivo*–a method for functional characterization and labeling of neurons in macaque V1. J Neurosci Methods 156, 37–49, doi: 10.1016/j.jneumeth.2006.02.004 (2006). 16540174

[b40] Gold C., Girardin C. C., Martin K. A. & Koch C. High-amplitude positive spikes recorded extracellularly in cat visual cortex. J Neurophysiol 102, 3340–3351, doi: 10.1152/jn.91365.2008 (2009). 19793873 PMC2804433

[b41] Sileo L. . Electrical coupling of mammalian neurons to microelectrodes with 3D nanoprotrusions. Microelectron Eng 111, 384–390, doi: 10.1016/j.mee.2013.03.152 (2013).

[b42] Rizzoni G. Fundamentals of electrical engineering. (McGraw-Hill, 2009).

[b43] Scorza C. A. . Morphological and electrophysiological properties of pyramidal-like neurons in the stratum oriens of Cornu ammonis 1 and Cornu ammonis 2 area of Proechimys. Neuroscience 177, 252–268, doi: 10.1016/j.neuroscience.2010.12.054 (2011). 21215795

[b44] Khoutorsky A., Heyman A., Shoseyov O. & Spira M. E. Formation of hydrophilic nanochannels in the membrane of living cells by the ringlike stable protein-SP1. Nano Letters 11, 2901–2904, doi: 10.1021/nl201368w (2011). 21651305

[b45] Spira M. E., Oren R., Dormann A. & Gitler D. Critical calpain-dependent ultrastructural alterations underlie the transformation of an axonal segment into a growth cone after axotomy of cultured Aplysia neurons. J Comp Neurol 457, 293–312, doi: 10.1002/cne.10569 (2003). 12541311

[b46] Spruston N. & Johnston D. Perforated patch-clamp analysis of the passive membrane properties of three classes of hippocampal neurons. J Neurophysiol 67, 508–529 (1992). 1578242 10.1152/jn.1992.67.3.508

[b47] McAdams E. T., Jossinet J., Subramanian R. & McCauley R. G. Characterization of gold electrodes in phosphate buffered saline solution by impedance and noise measurements for biological applications. Conf Procs IEEE Eng Med Biol Soc 1, 4594–4597 (2006). 10.1109/IEMBS.2006.260406 17946639

