# Supplementary material for: Multisite electrophysiological recordings by self-assembled loose-patch-like junctions between cultured hippocampal neurons and mushroom-shaped microelectrodes
Source: Sci Rep. 2016 Jun 3;6:27110. doi: 10.1038/srep27110 (PMC4891817; doi:10.1038/srep27110)
Supplement: Supplementary Information [file srep27110-s1.pdf]

Supplemental Material *

Multisite electrophysiological recordings by self-assembled loose patch-like junctions between cultured hippocampal neurons and mushroom-shaped microelectrodes

Nava Shmoel[1#], Noha Rabieh[1#], Silviya M. Ojovan[1], Hadas Erez[1], Eilon Maydan[1], and Micha E. Spira[1*]

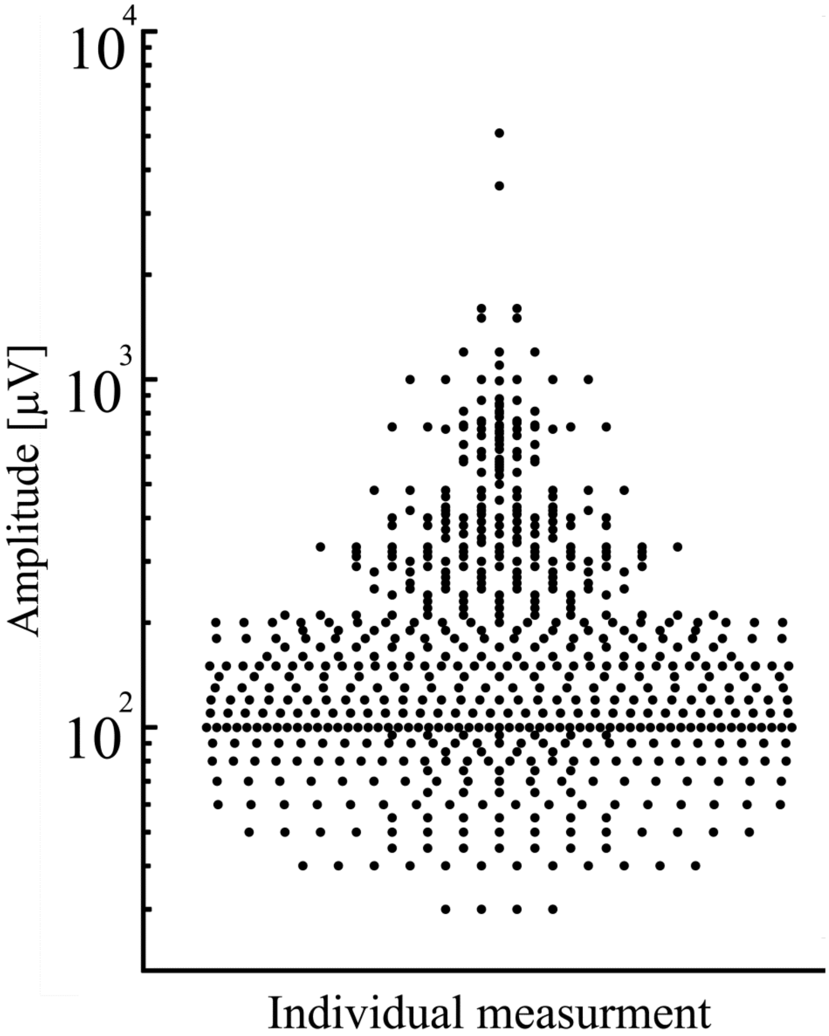

Supplementary Figure 1. Individual measurements of the peak amplitudes (logarithmic scale) recorded by gMµE-MEAs (number of MEAs=46, number of neurons=695).

Multisite electrophysiological recordings by self-assembled loose patch-like junctions between cultured hippocampal neurons and mushroom-shaped microelectrodes

Nava Shmoel[1#], Noha Rabieh[1#], Silviya M. Ojovan[1], Hadas Erez[1], Eilon Maydan[1], and Micha E. Spira[1*]

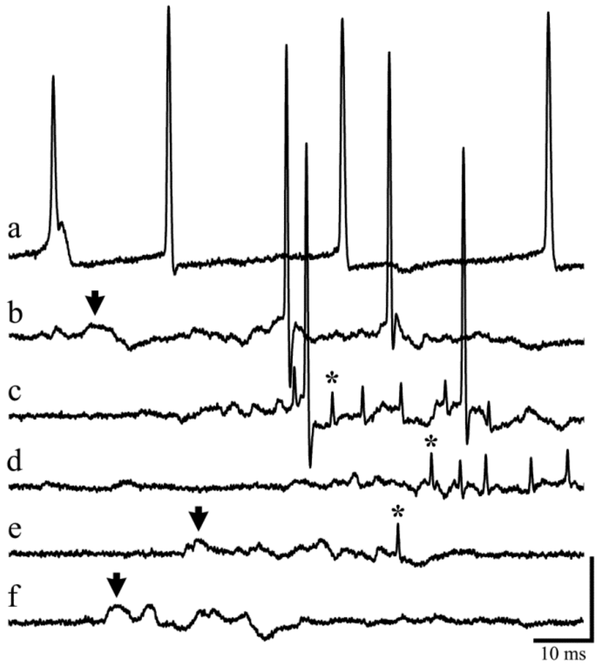

Supplementary Figure 2. Large spikes, small spikes (asterisks) and slow "ripples" (arrows) can be recorded by a single gMμE. The spikes (large and/or small) and the ripples can be recorded concomitantly (c) or separately. (a) only large spikes and (f) only ripples. Since either large (a) or only small spikes (d) can be recorded, it is reasonable to assume that the large and small action potentials are generated by two different neurons that interface with the same electrode. The ripples could reflect the firing of remote neuronal clusters or alternatively represent a barrage of synaptic potentials. For additional details see text. (a) Large action potentials abruptly "take off" from the baseline of the trace. (b) Concomitant recordings of membrane ripples and large action potentials alone. (c) Membrane ripples large action potentials and a burst of small action potentials. (d) Ripples and small action potentials alone. (e) Ripples and a single small action potential. (f) Only ripples.

Multisite electrophysiological recordings by self-assembled loose patch-like junctions between cultured hippocampal neurons and mushroom-shaped microelectrodes

Nava Shmoel[1#], Noha Rabieh[1#], Silviya M. Ojovan[1], Hadas Erez[1], Eilon Maydan[1], and Micha E. Spira[1*]

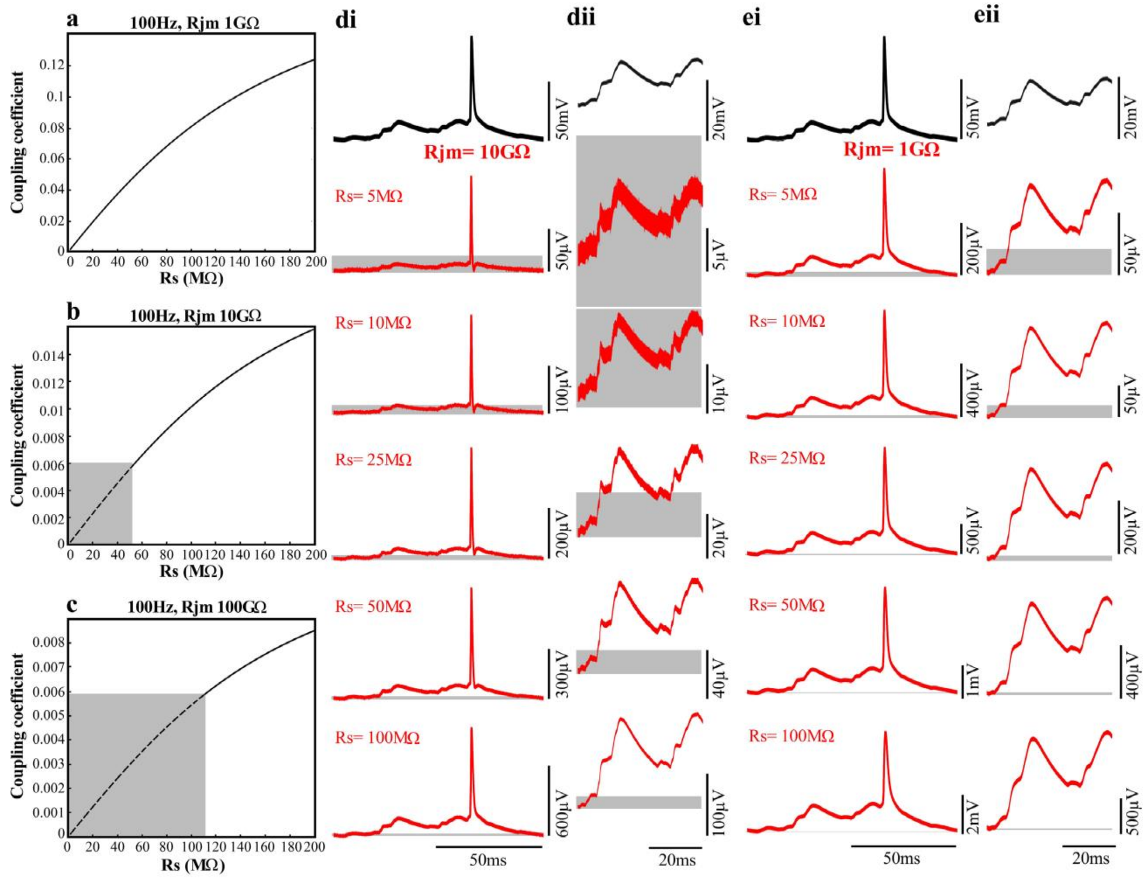

Supplementary Figure 3. Simulation of the electrical coupling coefficient of synaptic potentials as a function of the seal- (R_s) and junctional- (R_{jm}) membrane resistances. The simulation in (a), (b) and (c), was conducted by delivery of 100Hz sine wave depicting synaptic potentials to an analog electrical circuit (text figure 4) with R_{jm} values of 1 (a), 10 (b) and 100 GΩ (e). Assuming that: (i) the amplitudes of the synaptic potentials is 10 mV, (ii) that the noise level of the recording system is 20μV, (iii) that potentials with amplitude 3 times larger than the noise level (≥ 60 μV) can be detected. Then, theoretically coupling coefficient of ≥ 0.006 (60 μV) are sufficient to detect synaptic potentials. The gray areas and dashed lines in (b) and (c), mark the seal resistance values that prohibit recording of the simulated synaptic potentials. All other R_s values (white background) permit the detection of 100 Hz sine wave generated by a 10 mV source.

The expected recording quality as a function of the seal resistance is further illustrated by simulations of the "recorded synaptic potentials" (d) and (e). For the simulations, a trace of

patch electrode recording of an APs and a barrage of synaptic potential (black traces in (d) and (e)) was fed into a simulation circuit where R_{jm} was set to be 10GΩ and R_s values of 5, 10, 25, 50 and 100 MΩ (red traces in d,) or an R_{jm} of 1MΩ and R_s values of 5, 10, 25, 50 and 100 MΩ (red traces in e). Black traces (ei) and (di) depicts low gain recordings of an action potential and synaptic potentials. Black traces (dii) and (eii) are high gain recordings of the synaptic potentials only. The red traces depict the simulated recordings for the Rs values marked near the recorded potential. Note that for Rjm=10GΩ and R_s values of <50 MΩ (d) the expected synaptic signals are lower than the 20 μV noise level (gray background) and thus cannot be recorded. Nevertheless for Rjm=1GΩ synaptic potentials can be recorded even when R_s is as low as 5 MΩ (e). For all simulations shown R_{ep}=10 MΩ, CPE = 25 MΩ at 1 KHz and the amplifier impedance =20 MΩ at 1 KHz.

Multisite electrophysiological recordings by self-assembled loose patch-like junctions between cultured hippocampal neurons and mushroom-shaped microelectrodes

Nava Shmoel[1#], Noha Rabieh[1#], Silviya M. Ojovan[1], Hadas Erez[1], Eilon Maydan[1], and Micha E. Spira[1*]

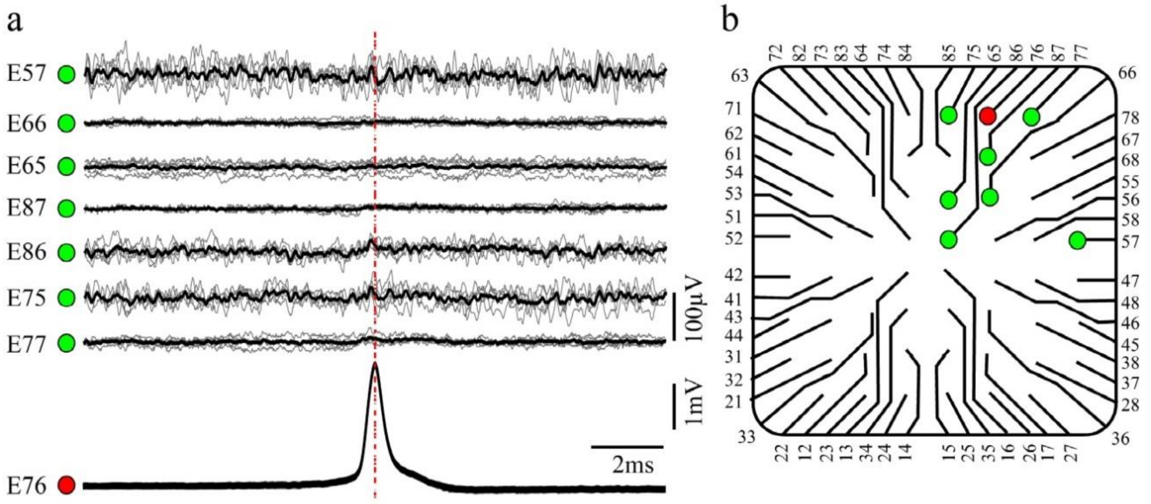

Supplementary Figure 4. An example of the approach to define the crosstalk levels between individual gMµE-MEA channels. The level of crosstalk between channels was assessed using 7-10 day old cultures. (a) Simultaneous voltage recording from the cluster of 8 gMµE shown in (b). Electrode E76 (red in (a) and (b)) recorded a >2mV monophasic action potential from a spontaneously and repeatedly firing neuron (the black trace in (a) is the average of five firing events). The average of simultaneously recorded voltage traces from 7 nearby electrodes (labeled green in (a) and (b)) are displayed at a higher gain above trace E76. There was NO crosstalk between the channel that recorded the AP and the cluster of the nearby gMµE channels (the small blip in E75, E86 and E57 is not crosstalk as can be clearly seen when enlarging the figure).

Multisite electrophysiological recordings by self-assembled loose patch-like junctions between cultured hippocampal neurons and mushroom-shaped microelectrodes

Nava Shmoel[1#], Noha Rabieh[1#], Silviya M. Ojovan[1], Hadas Erez[1], Eilon Maydan[1], and Micha E. Spira[1*]

Estimation of gMμE resistance and capacitance for simulation purposes

The impedance of freshly fabricated gMμE was measured using an HP 4284A Precision RLC meter, at 1 KHz, at room temperature, in a 0.9 % NaCl solution (Ojovan et al. 2015) [33]. The average values of a constant phase element (*CPE*) with a resistance of 3.5 MΩ and a capacitance of 5.1 pF in series were extracted. Impedance spectrum measurements revealed that newly fabricated gMμE have pure capacitive characteristics with an impedance that follows Z ∝ 1/f (where f is the frequency in the range of 1 to 100 kHz). Hence, the complex nature of the *CPE* could have been neglected for all the relevant frequencies and be presented as a simple passive element.

It is well-established that the impedance of gold electrodes can be reduced in a number of ways. Practically, exposure of gold MEA to plasma oxygen and ionic solutions leads to reduced impedance. This reduction can be depicted as an increased parallel conductance to the *CPE* (Fig. 4). To estimate the value of the parallel resistance (R_{ep}) we characterized the properties of the gMμE by applying a calibration voltage square pulse (10 mV, 10 ms) between an Ag/AgCl electrode immersed in the solution and the ground, and measured the voltage read by the MEA amplifiers before and after oxygen plasma and incubation of the gMμE in a phosphate buffer solution for 24 h (Fig. 4, a and b). As a result of these treatments, a substantial increase in the rate of rise and decay of the calibration pulse was recorded (defined by $\tau = RC$) and a higher saturation voltage was observed. Since the steady state output voltage is mainly determined by the ratio of the gMμE-R_{ep} to the amplifier input resistance, we concluded that the gMμE resistance was reduced. To estimate the R_{ep} of the gMμE we simulated the calibration pulse output for gMμEs of different R_{ep} in the range of 10-1000 MΩ in parallel to a *CPE* of 25 MΩ at 1 KHz [33].The simulation revealed that the observed experimental changes in the gMμE resistance were best simulated by changing the R_{ep} from a value of approximately 1 GΩ to 10 MΩ (Fig. 4, c).

To estimate the range of errors that can be introduced by using the wrong R_{ep} value for the simulations of action potential amplitudes we examined the frequency response of the system for values of R_{ep} between 10 MΩ and 100 MΩ (Fig. 4, d). The Δ line (in Fig. 3, d) represents the absolute difference between the two curves. Whereas at low frequencies (100-400 Hz) the error introduced is significant, at 1,000 Hz (representing action potentials) the difference is 200 μV or $\frac{200\mu V}{1.5mV} = 13\%$. We assume that the margin of inaccuracy of our estimate of R_{ep} is smaller

than one order of magnitude (10 MΩ compared to 100 MΩ), thus making our simulations accurate enough to estimate the properties of the gMµE.

Multisite electrophysiological recordings by self-assembled loose patch-like junctions between cultured hippocampal neurons and mushroom-shaped microelectrodes

Nava Shmoel[1#], Noha Rabieh[1#], Silviya M. Ojovan[1], Hadas Erez[1], Eilon Maydan[1], and Micha E. Spira[1*]

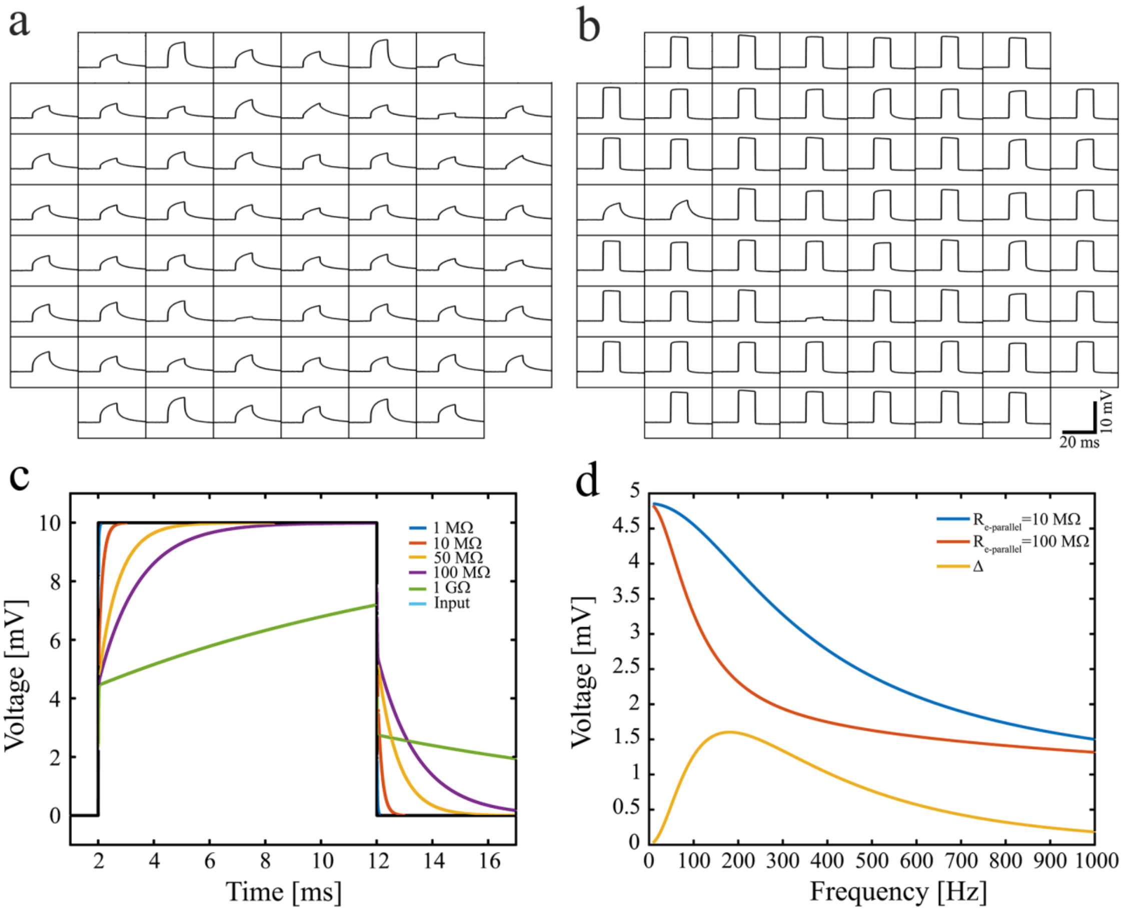

Supplementary Figure 5. Reduction of the gMµE impedance by plasma oxygen and incubation in an ionic solution (a, b) and estimate of the gMµE impedance. (a and b) Calibration voltage square pulse (10 mV, 10 ms) was delivered between an Ag/AgCl electrode immersed in the bathing solution and the ground. The recordings show voltage read by the gMµE-MEA amplifiers before (a) and after (b) oxygen plasma and incubation of the gMµE in a phosphate buffer solution for 24 h. (c) Simulation of the calibration output trace for gMµE with R_{ep} values ranging from1 MΩ to 1 GΩ. In black, the input voltage, and in color the values of $R_{ep.}$ (d) Frequency response of the system for R_{ep} values of 10 MΩ (blue) and 100 MΩ (orange). On the Δ line (yellow) the difference between the two curves. Note that at 1K Hz the difference is small.
